# HRH dimensions of community health workers: a case study of rural Afghanistan

**DOI:** 10.1186/s12960-019-0347-7

**Published:** 2019-02-06

**Authors:** Said Ahmad Maisam Najafizada, Ronald Labonté, Ivy Lynn Bourgeault

**Affiliations:** 1 Community Health and Humanities, Memorial University of Newfoundland, St. John’s, Canada; 2 School of Epidemiology and Public Health, University of Ottawa, Ottawa, Canada; 3 Telfer School of Management, University of Ottawa, Ottawa, Canada

**Keywords:** Community health workers, Human resources for health, Interprofessional relations, Afghanistan

## Abstract

**Introduction:**

There is ample evidence to indicate that community health workers (CHW) are valuable human resources for health in many countries across the globe, helping to fill the gap created by a chronic health workforce shortage. This shortage is not only in number but also in workforce distribution and skill mix. There remains a lack of evidence, however, concerning the size and distribution of CHWs and their relationship to the professionally regulated and recognized health workforce, such as physicians and nurses, and the unregulated and unrecognized health workforce, such as traditional birth attendants and traditional healers. This is particularly the case in low-income, under-resourced countries, such as Afghanistan.

**Method:**

We conducted a descriptive qualitative analysis involving fieldwork in Afghanistan between 2013 and 2014. We undertook participant observation and in-depth interviews with community members, CHWs, health managers, and policymakers, in an attempt to add more depth to our knowledge of how CHWs function, or could function better, as a recognized health worker.

**Results:**

We found that the number of CHWs has increased dramatically in recent years and that CHWs play a variety of roles, including work generally associated with professional providers, such as referral, education, and counseling. Although not a replacement for professional health providers, CHWs, in places where the number of and access to such providers is low, become the only option to meet basic health needs of the population. In places where professional providers are available, CHWs have the potential to extend the services to marginalized populations, provide community health services, and become a recognized member of the health provider team. A limitation of their role in health system strengthening is their lack of integration and a clear career path including into more recognized professional roles.

**Conclusion:**

CHWs provide a critical human resources for health role in Afghanistan, but there are opportunities for improved integration with other providers which can increase their potential to improve service delivery.

## Introduction

Most countries deploy community health workers (CHWs) to address a shortage or maldistribution of professional and regulated human resources for health (HRH) such as physicians, nurses, and midwives [1, 2]. CHWs are members of a community who are trained and supported by the health system to provide basic services in the communities in which they live [3], and are a valuable cadre in the health workforce in many countries across the globe [1, 4–7]. The overall number of CHWs was reported to be around 700 000 in India [8], 100 000 in Bangladesh, 30 190 in Ethiopia, and around 240 000 in Brazil [9]. Yet, the shortage of HRH is not only about the number but also about the geographic distribution, skill mix, and career paths of the workforce. Despite a general agreement that CHWs are a valuable health workforce, they are often viewed as elements of health programs rather than standalone human resources for health. In fact, there is a hesitance among some policymakers to recognize CHWs as a formal HRH, which in turn positions them implicitly as second-grade health service providers [10].

Despite the extensive use of CHWs across the globe and a growing evidence base for their training and the types of services they provide [11], there is a lack of contextual evidence on the size and distribution of CHWs, their career path, and their relationship with the professionally regulated and recognized health workforce as well as to other unregulated and unrecognized health workers such as traditional birth attendants and traditional healers. This is perplexing as CHWs often interact frequently with both, and the interaction has impact on the tasks of CHWs. Understanding the size of CHWs within a geographic context implies the shortage of professional providers and refers to CHWs importance as service providers [6]. The distribution of CHWs can be associated with the geographic distribution of health services in a context. In this article, we focus on a single case-study of Afghanistan, where a large number of CHWs have been recruited, trained, and deployed to address a chronic shortage of HRH.

Afghanistan faces a chronic shortage of skilled health professionals (9.4 per 10 000 population). The country has only 1.9 physicians for 10 000 people distributed unequally across the country with a rate as high as 7.2 in urban areas, and as low as 0.6 in rural areas [2]. The rest of the skilled health workforce included in the report by Global Health Workforce Alliance and WHO is comprised of nurses, midwives, and dentists [2]. On average, there are four times more skilled health professionals in urban areas than in rural areas [12]. According to the Global Health Workforce Alliance and WHO (2013), the density threshold of 22.8 skilled health workers per 10 000 people is required for most countries in need to achieve relatively high coverage for essential health interventions. Afghanistan is one of the countries with a least likely chance of hitting the threshold of 22.8 skilled health workers in 2035 [2].

Despite this shortage, the Afghan health system claims to cover 60% of its population for basic health services [13], with CHWs as one of the key first points of contact with the health system, with a focus on maternal and child health. In 2003, the Afghan government with the support from international donors initiated a national initiative called the Basic Package of Health Services (BPHS), which included training volunteer CHWs in rural areas to expand basic health services. CHWs have taken on the task of providing health services to the rural communities, bringing community members closer to the formal health system to which they previously had little access. The BPHS policy does not recognize the categories of traditional birth attendants, healers, and Hakimjis,^1^ and CHWs are not supposed to have any collaboration with them. The program was funded by the World Bank, the United States Agency for International Development, and the European Union, and contracted out to national and international non-governmental organization for implementation. By 2014, around 26 000 male and female CHWs were trained across the country. An earlier article from this study affords a detailed description of the Afghan CHW program, highlighting issues of training and recruitment, task, supervision, and compensation [14].

### Objective

Our research objective in this paper is to offer a descriptive qualitative analysis of how CHWs function as human resources for health in rural Afghanistan, and how they interact with both formal and informal health workers in the Afghan health system. We compare HRH dimensions of CHWs with other formally recognized HRH, including physicians and nurses, and the unrecognized workforce such as traditional birth attendants (TBAs) and traditional healers. The specific HRH dimensions we examine focus on the [1] size and distribution, [2] skills, [3] relationship with other formal and informal health workforce, and [4] CHW career paths.

## Methods

We undertook a descriptive qualitative analysis involving fieldwork in Afghanistan between 2013 and 2014, collecting data and conducting interviews with community members, CHWs, health managers, and policymakers. Participants were selected using stratified, purposive sampling, a method that divides the population into separate subgroups and then creates a sample by drawing subsamples from each of those subgroups. Stratified sampling ensures that all subgroups within a population are potentially represented in the sample, and purposive sampling ensures that stratified sampling is systematically implemented [15]. Since purposive sampling was applied, record of the proportion of non-participants was not kept systematically. The population for this research was stratified hierarchically and horizontally (Table 1). Hierarchically, they were divided at a policy level, management level, and community level. Horizontally, policymakers were stratified into government, international agencies and donor agencies; implementing organizations were stratified into international NGOs, national NGOs, and provincial health departments; and communities were stratified into less remote and high remote areas where the CHW program was implemented.

**Table 1:** Hierarchical and horizontal classification in sampling

Hierarchical	Horizontal		
Policy - Policymakers	Government	Donor agencies	UN agencies
Management	Public health departments	International NGOs	National NGOs
- Managers			
- CHW supervisors			
- CHW trainers			
Community	Less remote communities		Highly remote communities
- CHWs			
- Community members			

In-depth interviews were conducted with policymakers in Kabul, health managers of NGOs implementing the program in provinces, and CHWs and community members in villages. Policymakers were selected based on their knowledge of the CHW program through consultation with Ministry of Public Health officials and researchers in Afghanistan National Public Health Institute. Once a number of potential participants were identified, the solicitation email was sent to them. Upon their agreement, a date and place were set with the participants and interviews were conducted. Most potential participants agreed to the interview. Lack of time was the main reason for some policymakers not participating in the study.

Health managers were selected based on the three types of implementing organizations (international NGOs, national NGOs, and provincial government health departments). First, the lead researcher contacted the implementing organizations and met with the director of the organizations to identify potential health managers who had good knowledge of the program. Then, the researchers sent a solicitation letter to the managers to ask them for their participation. All managers agreed to participate except one who expressed his lack of in-depth knowledge on the subject. The lead researcher traveled to each province to interview health managers in their offices at their own availability.

To recruit CHW participants, the lead researcher gathered telephone numbers of all active CHWs from implementing organization and tried to contact them ahead of time, explain to them the research, and request their participation. Most agreed. Only three CHWs contacted through phone did not participate stating “not interested and not available.” In some cases, when enough CHWs could not be contacted through their cellphones, the researcher, accompanied by a female research assistant, traveled to villages where the program was active, visited the health posts where CHWs worked, explained the research to CHWs, obtained verbal or written consent, and conducted interviews. A female research assistant led the way when only female CHWs were present at the health post. All CHWs approached personally at the health posts agreed to participate. Focus groups were also conducted with CHWs and community members at the villages (Tables 2 and 3).

**Table 2:** Participants

	Participants	Participants’ criteria	Number
Policymakers	Ministry of Public Health	Involvement in CHWs program design and implementation (Health Officers, Health Advisors, Community-based Health Care Department officers, Health Economics and Finance Department Consultants, Deputy Minister of Policy and Human Resources)	5
	USAID^1^		1
	World Bank		1
	European Commission		2
	DFATD^2^ - Canada		1
	WHO^3^		1
	UNFPA^4^		1
		Sub-total	12
Implementing organizations	Health managers	International NGO National NGO Provincial Health Department	8
	CHSs^5^		11
	CHW trainers		4
		Sub-total	23
Community	CHW community members		28
		8 focus groups	35
		Sub-total	63
		Total	98

^1^United States Agency for International Development

^2^Department of Foreign Affairs, Trade and Development

^3^World Health Organization

^4^United Nations Population Fund

^5^Community Health Worker Supervisors

**Table 3:** Additional information on data collection

First round of data collection	Second round of data collection	
	Member checking	Interviewing/member checking with new participants
55 individual interviews	18 member checking	8 interviews/member checking
8 focus groups (25 people)		3 focus groups (10 people)

Across all categories, participants were recruited until there was saturation in the corroboration of the descriptive findings. Interviews and focus group guides probed for a number of facets of the CHW program and included specific questions about the tasks of CHWs compared to other providers, the relationship of CHWs with other professional and traditional providers, and potential career paths for CHWs.

Augmenting the interviews, field notes were taken during site visits to document observations of health facilities for their structure, distance from villages, wards, patients, services, staff, and equipment, of the villages for the sources of drinking water and the location and the type of latrines, electricity availability, distance from clinic, and main source of income. The final field notes included a journal of what happened during these visits, relevant statements, and the two researchers’ initial analytical reflections.

Interviews, conducted by the lead researcher and his assistant, were audio-taped and extensive field notes documenting observations made. The field notes were used to crosscheck or complement the interview data and to assist in contextualizing the interview analysis. A preliminary data analysis was conducted after phase 1 before the lead researcher traveled back to the field for member checking in the second phase of fieldwork. In this second round of fieldwork, the lead researcher shared the preliminary findings with previous participants, as well as with new participants for comment, confirmation, and further data gathering. Member checking is a validation procedure in qualitative methods adding value to the analysis of the raw data, as the participant takes part not only in providing the data but also in the interpretation of them. This process also helps to clarify misunderstandings, wrong information, and misinterpretations [16]. The second round took place between September and October 2014.

Interviews and focus groups were translated and transcribed by the lead researcher. Initial analysis began during the fieldwork, consisting of the researchers’ initial reflections and field notes. Final thematic analysis was carried out by manually coding the transcripts into nodes, which were then put into sub-themes and then broader themes using a constant comparison technique [17]. Representative quotes were selected based on the way they resonated with most respondents. The context in which each quote was expressed and what participant group made the statements are explained to enrich the analysis. All quotes in this paper have been anonymized by giving a code number to the broader participant groups.

Ethical procedures of consent, safety, confidentiality, and privacy were considered during data collection. The ethics review board of the University of Ottawa and Afghanistan’s National Public Health Institute approved the study.

## Results

The data was collected between July 2013 and November 2014. The lead author [MN] visited 17 villages, in 9 districts of four provinces. Overall, he interviewed 12 policymakers, 8 health program managers, 15 community health supervisors and trainers, 28 community health workers, and 35 community members, totalling 98 participants (Table 2). In total, 54 of the participants were male and 44 of them were female (Table 4).

**Table 4:** Gender of study participants

Key informants		Male	Female	Total
Community members	First phase	11	14	25
	Second phase	6	4	10
CHWs	First phase	9	16	25
	Second phase	2	4	6
Supervisor and trainer	First phase	9	4	13
	Second phase	2	0	2
Health managers		6	0	6
Policymakers		9	2	11
Total		54	44	98

### Volunteer CHWs: the issue of size, distribution, and motivation

Based on the Ministry of Public Health’s Health Management Information System’s database, the 26 000 volunteer, trained CHWs are by far the largest health workforce in the country, with 7.43 CHWs (compared to 1.9 physicians) per 10 000 populations. According to the BPHS policy, the catchment area for a health post was an average of 1250 persons (100 to 150 households). Our descriptive analysis of the Ministry of Public Health’s administrative database shows that in 2012 only 6 provinces (out of 34) had reached the standard of 1 health post for 1250 people. And as many as 8 provinces had one health post for around 2500 persons.

One of the main reasons for the considerable size of the workforce is the voluntary nature of the program. Our findings suggest that individuals who volunteer as CHWs are driven by several, intertwined motives. Altruism, social recognition, personal knowledge gain, and a desire to enter or climb up a ladder of health professionals (mainly community health supervisors, nurses or midwives, which are all paid positions) are a number of common motivators.

Based on our interviews with CHWs, the most common response regarding the reason CHWs volunteered was that they wanted to serve their community. A strong sense of community could be due to the tribal and communal structure of the Afghan society. Religious beliefs and the promise of reward in the next life were also mentioned as motivators for volunteerism.

We have committed to volunteer and want our community to prosper. If people appreciate our work, that’s better. (CHW #12, female)

CHWs in the Afghan program are deployed only in rural and remote areas, whereas professional providers congregate in urban settings. The distribution of CHWs in rural areas where there were often no other health providers make them valuable members of the overall health workforce. In response to how important CHWs were to their communities, a health policymaker remarked:

I would say [they are] more [important] than the Minister of Public Health, because they are at least available, and they do whatever they can, if they couldn’t do anything, they refer to a clinic or a hospital. (Policymaker #7, male)

In many of the villages visited for this research, CHWs were called “village doctors.” CHWs had the authority to distribute drugs, treat common childhood diseases such as pneumonia and diarrhea, provide counseling, and refer patients to health facilities.

CHWs also sought to gain new knowledge and skills, which they believed would not only be advantageous to the community but also to themselves and their families. Those CHWs in their 20s or younger hoped that working as a volunteer was a step to further training opportunities to become community health supervisors, midwives, nurses, or even medical doctors. These motivations are particularly strong in female CHWs, who account for almost half of the CHWs. They are reportedly more active than their male counterparts (who provide sanitation and environmental programs) and are the major providers of maternal and child health services in rural communities. Having had the opportunity to learn, gaining higher social status in their families and communities, and a prospect for future training and employment gave these female CHWs a sense of empowerment. Moreover, working as a CHW provided the opportunity for women to move around in their village and socialize with other women.

CHWs participating in this study reported an average work experience of around 6 years, ranging from CHWs who had worked in the program since its inception in 2003, to CHWs newly trained to replace the dropouts (2% according to the CBHC databases, August 2014). Most health managers and policymakers believed the dropout rate could have been higher but not so high to challenge the efficiency of the entire program. The stated common reasons stated for dropout were moving out of their community, marriage for the women, and income opportunities for men. CHWs received monetary incentives such as travel expenses, lunch money, and educational stipends, and non-monetary incentives such as stationary for a community map, toothbrush, toothpaste, and hand soap. Health managers and policymakers noted that those monetary and non-monetary incentives provided by the health system were important factors in CHW recruitment, deployment, and retention.

### Skill mix: CHWs unique combination of skills

We found that besides the assigned tasks CHWs had developed some practical skills to achieve the health outcomes in their communities. They often provide services in pairs, undertake some tasks of the professional providers, and have their own specified tasks.

#### Group providers

CHWs work in pairs providing a mix of many different services compared to other health professionals who worked alone. The pair most often includes a man and a woman, each undertaking gender-appropriate health tasks ascribed to each gender by the community to better serve people living in his or her catchment area. Noteworthy is that female CHWs were more active, as they were ascribed the majority of the tasks related to maternal and child health, which was the focus of the overall CHW program.

#### Shifted tasks

A basic health center in rural Afghanistan generally includes a physician, a nurse, a midwife, a pharmacist, a laboratory technician, and an administrator. In a Health Post, one male and one female CHW undertake a combination of the gender-specific tasks that overlap with of all of those health providers in a basic health center. CHWs detect signs and symptoms of prevalent diseases such as diarrhea and pneumonia and prescribe basic drugs such as co-trimoxazole (a combination of trimethoprim and sulfamethoxazole)(t) similar to a physician; dispense some drugs and advise on the usage of those drugs similar to a pharmacist; care for the sick, provide first-aid, and administer injections similar to a nurse; keep an eye on pregnant women and sometimes help with their deliveries similar to a midwife; and keep track of their own activities and the status of health in the communities similar to a health administrator.

If I go to the clinic for a headache, I get the same drug as a CHW would give me. (Community member, FG#3, female)

A small injury is treated similarly by a CHW and someone in the health facility, why trouble, [and] go the long distance! (Health manager #2, male)

The tasks shifted from professional providers to CHWs are simple, which may not require in-depth medical knowledge or experience.

#### Unique tasks

There were a number of other tasks that distinguish CHWs from professional providers. For example, CHWs are expected to refer complicated cases to a health facility, but knowing when to refer could be a complex task requiring a certain level of skill and knowledge. For example, the recommended practice for children with frequent and fluid defecation is oral rehydration solutions, while most children with a cough and fever are prescribed a single dosage of co-trimoxazole (t). If the treatment does not work, they are referred to the health facility. The reality is not that simple. Emphasizing the complexity of referral, a supervisor remarked:

What about the child who coughs and has fluid defecation? What about the child with frequent, little defecation? What about the child with a dry cough and without fever? What about the child with a cough with sputum? There could be many other variants of diarrhea and pneumonia. (Supervisor #4, male)

Referral requires knowledge and skills to distinguish between simple pneumonia and diarrhea, and complicated cases. The referral also depends on how vocal the patient is about her or his illness. The referral also depends on social conditions, such as availability of transportation, road conditions, weather, distance, patient or family’s decision regarding the referral, or lack of facilities to refer to. CHWs have gradually learned to deal with many of these situations.

Other important tasks that CHWs undertake, and which other health professionals in the health facilities often ignore, are health education for individual hygiene and behavior, and community mobilization for societal wellbeing.

Dispensing contraceptives is an effortless task but convincing individuals to actually use them is a difficult one. (Health manager #6, male)

Similarly, study participants noted that distributing soaps did not mean that the people would actually use them. One CHW said that telling people that to cover their drinking water source to keep it safe was one thing, but having them participate in actually covering water wells was another, or that asking people to build latrines far from their houses was one thing, while mobilizing community members to do so presented a different challenge. Acquiring skills and experience in community education and mobilization distinguishes CHWs from most other professional health providers.

The roles that CHWs have undertaken in the Afghan context, including the assigned ones and the ones that they have learned contextually, indicate their capacity to adapt to a changing context and to learn the tasks that are required there. Facilitating transportation under the role of referral, changing behaviors under the role of health awareness, and mobilizing community under the role of public sanitation are a few of the examples gleaned through interview data and observational field notes.

### CHWs’ relation with other health workers

CHWs in Afghanistan are essentially situated between professional and traditional health workers, placing them in an ambivalent yet potentially important place.

#### CHW-professional worker relations

CHWs in Afghanistan, in accordance with BPHS policy, are not directly linked with professional health workers such as nurses or doctors, although they were a source of referral to such professionals. They are instead linked with community health supervisors. A challenge arose when CHWs prescribed drugs for diarrhea, pneumonia, and fever (a common sign of infection) but did not receive feedback from a professional worker to know whether or not they had done their tasks appropriately. A community health supervisor could not determine whether the treatment or prescription of a CHW was medically correct. When a patient did not get well with the drugs provided by a CHW, he or she was referred to the health facility. CHWs only got feedback from the patient in a follow-up visit.

We see what kind of drugs the physician provided a particular patient and learn to give the same drugs to the same patient next time. (CHW #7, female)

Neither the trainers nor the supervisors of CHWs are professional health workers. The findings in this section have been limited due to a programmatic lack of relationship between CHWs and professional providers.

#### CHW-traditional workers relations

We found through field observations that in some villages where the CHWs work, traditional health workers such as religious healers, Hakimji, and traditional birth attendants already existed.

In some research sites in Afghanistan, traditional workers found the CHW program conflicting with their practices. For example, we were told that some religious healers would never promote contraception, or deny the existence of evil spirits that cause sickness.

I lost ten newborns. There were not doctors or nurses; our relatives were only using gunfire^2^ because they thought that my illness was only an Awla [Possession by an evil spirit], or religious healers were called to take care of it. (Community member, FG#3, female)

In 2015, the Afghan government invited 500 religious leaders for a conference in Kabul to encourage them to promote family planning.^3^

Some traditional birth attendants became fierce rivals of CHWs in the villages because they lost their main clients—pregnant women—to the CHWs in this new system. In one village in the central province of Bamyan where two female CHWs ran a Health Post, there were two other traditional birth attendants, who created problems for CHWs. A CHW noted.

They are mad at us; they say we have stopped their business. (CHW #8, Female)

Our observations suggest that such rivalry between female CHWs and traditional birth attendants was common in most rural areas. Female CHWs visited pregnant women, provided them with nutrition supplements and iron tablets, encouraged them to visit health facilities, educated them on the risks of pregnancy, helped them prepare a birth plan, and promoted institutional delivery. Institutional delivery is argued to be much safer in Afghanistan compared to home delivery [18]. Traditional birth attendants thus lost their clients and started preaching against female CHWs and health facilities. The most common false accusations made by traditional birth attendants and other anti-modern health facility elements were [1] the contraceptives would make women infertile, [2] there were no female providers in the health facility, and [3] health facilities were run by infidels. The rumors on contraceptives were further boosted by an emphasis of the side effects of the contraceptives:

Those who have used injectable contraceptives have grown fatter … and those who use pills get nervous … easily irritated, dizzy, nauseous, and have bleeding. (CHW #5, female)

In one site, our observations suggested that the side effects of the contraceptives helped the Unani/Greek medical providers in the local market. As CHWs would convince the villagers to give space between pregnancies, the negative side effects of the hormonal contraceptives led the villagers to Unani providers for herbal contraceptives.

People don’t use a lot of contraceptives due to their side-effects, they get herbal medicine from bazaar … A few people have used it, and they are happy with it … It is called herbal contraceptives from Aatay Jaffar [Jaffar’s Father’s Herbal Medicine] in the Titanic bazaar. (CHW#6, female)

Aside from false accusations and overemphasis on the negative, we learned from the site visits that the traditional health providers took advantage of some rumors against health facilities and CHWs. The common ones were that [1] the providers at the health facility did not behave well with patients, [2] that women were seen by a male stranger which is against Islamic law, and that [3] their mothers and grandmothers had given birth at home, and they turned out to be totally fine.

### Career path

We found that some CHWs in their 20s or younger hoped that working as a volunteer was a step to further training opportunities to become community health supervisors, midwives, nurses, or even a medical doctor. A female CHW in Bamyan Province said:

I was told to be accepted in the midwifery program if I volunteered as CHWs.

In fact, some CHWs have gone to become community health supervisors, nurses, and midwives. Many of the supervisors we interviewed in our study had experience as CHWs. Despite the fact that some CHWs have become paid health workforce, the Afghan CHW program does not have a standard career development route. First, there are many CHWs who cannot read and write, hindering further training and career opportunities for them. Second, a lack of accreditation and licensing procedure for CHWs limits their career opportunities. The Afghan Ministry of Public Health human resource policy provides a definition for health worker certification,^4^ accreditation,^5^ and licensure.^6^ All categories of professional health workers are trained in educational institutes administered or accredited by the Ministry of Higher Education. CHWs, however, are trained by the BPHS implementing organizations and provided with a certificate approved by the Ministry of Public Health. The Ministry of Public Health in collaboration with the WHO developed the CHW training curriculum without the involvement of the Afghan Ministry of Higher Education, the entity that accredits educational institutes and programs in Afghanistan. CHWs needed standard training module approved by the Afghan Ministry of Higher Education in order to go through the process of accreditation and licensing. The lack of this approval means that CHWs cannot get into advanced educational programs administered and accredited by the Ministry of Higher Education solely with their CHW training and experience.

Third, there are not many community-based careers to which CHWs might advance. During site visits, CHWs said that they were told they would have opportunities to become nurses, midwives, and supervisors. Some female CHWs did receive further training and became midwives, and some CHWs ended up becoming supervisors. Our observations indicate that having a high school education (rare for most CHWs) was a significant factor in CHW admission into midwifery training. Midwifery programs operate independent of the CHW program and do not have CHW training or experience as a prerequisite or preferable criterion for admission [12, 19]. In addition, a number of health managers noted that they preferred male CHWs to become supervisors when there was a job opening. Generally, however, it is only infrequent that CHWs are promoted to paid jobs as supervisors or midwives.

## Discussion

In this study, we found that volunteerism has played a major role in deploying a large number of CHWs in Afghanistan. CHWs undertake a variety of roles including tasks shifted from professional providers such as treating common illnesses such as headache, diarrhea, and pneumonia, making referrals within the health system, providing health education, and initiating community mobilization efforts. CHWs do not have a direct relationship with professional providers, and they negotiate relationships with traditional providers contextually. CHW’s lack of integration into the country’s education system of the human resources of health and a lack of a clear career path hinder their struggle towards a more recognized professional role within the health system.

CHWs are used all over the world to help address HRH shortages, particularly in underserved areas [1, 14, 20]. The addition of CHWs may increase the ratio of human workforce per population, but their inequitable distribution remains a problem [21, 22]. CHWs are inequitably distributed among the marginalized, the poor, and rural and remote populations [1, 23–25], with consequences for the workers and the populations the serve. Findings from our study largely affirm these outcomes from other studies. With 26 000 CHWs in Afghanistan, CHWs have been able to address the shortage of human resources, at least to some extent, and have become the health workforce for the poor. With the current CHW program in Afghanistan, the population reached by the program has received minimum health service, while the government claims a large coverage for CHW services.

With regard to their skills, CHWs are a unique type of human resources in comparison with other established cadres. The tasks of CHWs all over the world are numerous but it is their flexibility that gives them an advantage. In the case of Afghanistan, their tasks range from detection and treatment of prevalent diseases, to health education and awareness, to referral, and to administration of a Health Post, which were all based on the needs of the communities [14]. In the recent outbreak of Ebola virus in the West Africa and Nigeria, CHWs were the frontline workers to engage with communities limiting the spread of the virus [26]. With increasing new cases of polio in Uttar Pradesh, India, in 2011, CHWs contributed to the elimination of polio transmission there [26]. In Afghanistan, professional health providers, who are the main HRH of health systems, do not supervise CHWs, leaving CHWs with a feeling of isolation from the health system [14]. Their programmatic contributions can be best utilized when CHWs are made members of a primary health care team rather than left alone or isolated from other health workers.

Another characteristic that makes CHWs particularly useful within health systems is the unique location that CHWs occupy, navigating between formal and informal HRH systems and crossing boundaries between the two [27]. As volunteers, they are situated within the community and the informal health systems. As health workers, they are viewed as members of the health system. As a member of the health system, they attempt to gain a status within the health system, perhaps as a member of the health providers’ team [20, 27]. In Afghanistan, CHWs have been able to use their referral power and links with the formal system to bridge the western health care with traditional communities in the rural and remote areas.

One of the spillover effects of the CHW program in Afghanistan has been a disruption in the gender dynamics of communities they serve. Half of the 26 000 CHWs in Afghanistan are women. The large deployment of female CHWs has the potential to affect the distribution of health resources, division of health labor, gendered social norms, and decision making on health issues in local communities [10].

Researchers have long argued that CHWs should not be considered a panacea for weak health systems [28, 29]. Whereas CHWs may address a shortage of HRH to some extent, provide the skill mix necessary in complex settings, and navigate between the formal and informal heath systems, the bigger challenges of governance and management, financing, pharmaceuticals, and technological adoption in health systems must be addressed. In fact, strengthening weak health systems is more important for the better use of CHWs than the existence of CHWs to strengthen weak health systems. A stronger health system can pave a systematic career path for CHWs, which is the missing HRH dimension not only in Afghanistan but also in many other settings [30, 31]. A professional medical career path is not feasible and probably not necessary. CHWs can be promoted to positions of trainer, supervisor, and probably health managers.

Like all research, this study had some limitations. Professional providers and traditional healers were not interviewed as part of the research, and thus their perspectives on their relationship with CHWs were not investigated. The personal security of the researcher, assistant, and key informants was a key concern, limiting the places to which site visits could be made and limiting the range of interviewees, notably at the community level. Finally, participants’ responses may have been subject to social desirability or other biases, an issue commonly encountered in qualitative research. In our study, the principal researcher is a male, an ethnic Hazara, and a physician with additional foreign-training which may have influenced data collection from participants who were female and/or had a different ethnicity and social status. The researchers were aware of these potential biases and took efforts to minimize them by hiring a female research assistant for data collection, striving for a balance of ethnic backgrounds among participants, and continually de-briefing with the local researcher and the research supervisors over any potential evidence of bias in the study process and interpretation of results.

## Conclusion

CHWs in Afghanistan are a viable option to meet the basic health needs of the population, but their full integration into the health system as human resources for health is limited. The limitation to CHW integration and effectiveness comes partly from the fact that they are viewed as second-level providers. In places where professional providers are available; CHWs have the potential to extend the services to marginalized populations, provide community health services, and become a member of the health provider team. Most importantly, CHWs also have the capacity to assume new roles quickly, with the potential to help a complex health system adapt to changing contexts and needs. Attention to career progress is needed.

## Abbreviations

BPHS: Basic Package of Health Services
CHWs: Community health workers
HRH: Human resources for health
NGO: Non-governmental organizations
TBA: Traditional birth attendants

## Footnotes

1 Hakimjis are traditional health practitioners who use Greco-Arab and Unani [Meaning Greek in Persian] medicine. They are usually found in small bazaars in rural areas, and work out of a shop of herbal medicine. According to Unani medicine, health is considered as a state of body with humors in equilibrium and body functions normal. Health is based on six essential elements: (1) Air, (2) Drinks and food, (3) Sleep and wakefulness, (4) Excretion and retention, (5) Physical activity and retention, (6) Mental activity and rest

2 When a pregnant woman have difficulty delivering a baby, communities think she might be possessed by evil spirits, and fire gunshots to scare the evil spirit and make it leave the woman.

3 In August 2015, the United Nations Population Fund and the Afghan Ministry of Hajj and Endowment invited 500 religious leaders from across Afghanistan to promote family planning. http://afghanistan.unfpa.org/news/afghan-religious-leaders-spread-word-family-planning

4 Acknowledgement of those quality standards has been met. (e.g., certificate issued to indicate successful completion of a course of study having achieved the required levels of competence).

5 The process of external quality review, which is applied to training institutions and or programs to assess the quality. It measures the institution and or program against agreed upon standards thereby assuring that they meet the national agreed upon standards.

6 The approval given to an individual professional practitioner once minimal requirements have been met. This is obtained through examination (e.g., Testing and Certification Board Examinations) or through graduation from an accredited training institution.
